# Depression and Anxiety Outcomes Associated with Failed Assisted Reproductive Technologies: A Systematic Review and Meta-Analysis

**DOI:** 10.1371/journal.pone.0165805

**Published:** 2016-11-11

**Authors:** Adriana Milazzo, George Mnatzaganian, Adam G. Elshaug, Sheryl A. Hemphill, Janet E. Hiller, on behalf of The Astute Health Study Group

**Affiliations:** 1 School of Public Health, The University of Adelaide, Adelaide, South Australia, Australia; 2 College of Science, Health and Engineering, La Trobe Rural Health School, La Trobe University, Victoria, Australia; 3 Menzies Centre for Health Policy, The University of Sydney, Sydney, New South Wales, Australia; 4 Learning Sciences Institute Australia, Faculty of Education and Arts, Australian Catholic University, Melbourne, Victoria, Australia; 5 School of Psychology, Faculty of Health Sciences, Australian Catholic University, Melbourne, Victoria, Australia; 6 School of Health Sciences, Swinburne University of Technology, Melbourne, Victoria Australia; VU medisch centrum, NETHERLANDS

## Abstract

**Objective:**

Our study examined the psychological outcomes associated with failed ART treatment outcomes in men and women.

**Search Strategy:**

A systematic search for studies published between January 1980 and August 2015 was performed across seven electronic databases.

**Inclusion Criteria:**

Studies were included if they contained data on psychosocial outcomes taken pre and post ART treatment.

**Data Extraction and Synthesis:**

A standardised form was used to extract data and was verified by two independent reviewers. Studies were meta-analysed to determine the association of depression and anxiety with ART treatment outcomes. Narrative synthesis identified factors to explain variations in the size and directions of effects and relationships explored within and between the studies.

**Main Results:**

Both depression and anxiety increased after a ART treatment failure with an overall pooled standardised mean difference (SMD) of 0.41 (95% CI: 0.27, 0.55) for depression and 0.21 (95% CI: 0.13, 0.29) for anxiety. In contrast, depression decreased after a successful treatment, SMD of -0.24 (95% CI: -0.37,-0.11). Both depression and anxiety decreased as time passed from ART procedure. Nonetheless, these remained higher than baseline measures in the group with the failed outcome even six months after the procedure. Studies included in the narrative synthesis also confirmed an association with negative psychological outcomes in relation to marital satisfaction and general well-being following treatment failure.

**Conclusion:**

Linking ART failure and psychosocial outcomes may elucidate the experience of treatment subgroups, influence deliberations around recommendations for resource allocation and health policy and guide patient and clinician decision making.

## Introduction

Assisted reproductive technologies (ART) have become an important option for those seeking help to conceive [1] and are well-established [2–4] with increasing utilisation [5–6]. Despite increases in treatment usage, ART success rates, conventionally defined and measured as the rate of live births per cycle initiated [6] although improving are still modest [7]. In Australia, the live birth rate per treatment cycle was 17.9% in 2012 [5] with international data reporting rates approximately 2% higher [6]. There is a distinction in the reporting of success rates associated with frozen versus fresh autologous cycles. For example, in Australia rates per live delivery are 2% higher for women undergoing autologous thaw cycles than for autologous fresh cycles [5].

Factors related to lower success rates have been associated with duration of infertility, increasing number of ART cycles and increasing maternal age [8–12]. With increasing women’s age the live delivery rate per thawed embryo declines; this is similar if using autologous fresh embryos [12, 5].

The impact of ART failure on a person’s psychosocial state has not been considered when assessing treatment safety and effectiveness, nor has it been included in the evidence base when making decisions on policy directives around access to treatment [11]. By contrast, the potential for psychosocial gains through ART success or even through ART attempt does feature. ART clinicians perceive the positive psychological aspects of undergoing treatment rather than considering any potential adverse psychological outcomes associated with failure [13]. ART success shows marked differential effectiveness in treatment outcomes with parental age [9–11]. Internationally, women over 40 years of age are increasingly undergoing ART [14]. Evidence from Australia and New Zealand for example reveals that the fastest growing age group undergoing In Vitro Fertilisation (IVF) is women aged 40 or more. This age group proportion has been rising steadily from 14.3% in 2002 and 21.4% in 2007 to 25.3% in 2012 [5].

Females and males entering ART programs have high expectations of achieving a successful outcome which may result in disappointment if ART treatment fails [15]. A systematic review [16] evaluated psychological adjustment toIVF and found that overall women adjusted well to unsuccessful treatment cycles, as have some other studies which have concluded no change or positive emotional adjustment following failure [17–18]. In spite of this, numerous studies have found that women and men experience negative psychological outcomes after unsuccessful treatment [19–28]. With an increasing number of couples seeking treatment, including older couples, it is important to assess potential psychological impacts associated with failed treatment and to have this evidence incorporated in health policy deliberations on funding, access, and eligibility. To date, study results have not been pooled to quantify the effect of treatment failure by comparing psychological scores pre and post ART treatment. This systematic review and meta-analysis examines the psychological outcomes associated with failed ART. Intracytoplasmic Sperm Injection (ICSI) and IVF are the most widely used ART procedures worldwide and are thus the focus of this systematic review [5–6].

## Methods

### Search strategy

A systematic search for studies published between January 1980 and August 2015 was performed across seven electronic databases: MEDLINE, PsycINFO, CINAHL, Informit Health, Cochrane Library, Current Contents Connect and EMBASE. The search was limited to 1980 onwards in order to capture data from the beginning of the ART experience, as the first live baby born as a result of a successful ART treatment was in 1978 [29]. Search strategies were used in combination to identify all relevant psychological burden and psychosocial outcomes associated with studies with unsuccessful ART treatment. Specific terms searching for studies reporting successful ART treatment were not employed; rather, we included successful ART treatment outcomes if they were included in studies that reported psychosocial outcomes associated with failure.

Variations of the terms reproductive medicine OR assisted reproduct*OR in vitro fertil* OR sperm inject* AND treatment failure AND anxiety OR depression OR emotions OR adaptation, psychological were included (Table 1). The citation lists of relevant publications, reviews, and included studies were pearled for any relevant references not captured in the database searches.

**Table 1:** Search terms.

reproductive techniques, assisted [mh] OR reproductive medicine [mh] OR assisted reproduct* OR in vitro fertil* OR IVF OR sperm inject* OR ICSI
**AND**
treatment failure [mh] OR fail [tiab] OR failed [tiab] OR failure [tiab] OR failing [tiab] OR unsuccessful [tiab] OR unsuccessful cycles [tiab]
**AND**
anxiety [mh] OR anxiety [tiab] OR depression [mh] OR depression [tiab] OR psychological stress [mh] OR psychological stress [tiab] OR emotions [mh] OR emotions [tiab] OR adaptation, psychological [mh] OR adaptation, psychological [tiab] OR psychology, social [mh] OR psychology, social [tiab]

*Truncate free text term to search for variations.

### Study selection

#### Inclusion

Studies were included if they contained data on psychosocial outcomes that included, but were not limited to, marital and relationship satisfaction, emotional adjustment to failure or success including symptoms of anxiety, depression, and quality of life and emotional wellbeing. Emotional wellbeing related to psychological effects such as anger, happiness, confusion, and insecurity, sleeping difficulties, lack of self-esteem or self-confidence. Quality of life measures related to relationship difficulties with partner, to life satisfaction in general and to social desirability. Symptoms of depression and anxiety vary in severity and to be included in this study, measurements of both of these psychological outcomes needed to be well defined by use of operational definitions with valid and reliable scales [30]. For the meta-analyses, studies reporting either depression or depressive symptoms and studies reporting state or trait anxiety were included. Males, females, and couples with measured psychosocial outcomes before the start of an IVF or ICSI treatment cycle were compared to the same measures within the same population taken after treatment not resulting in a live-born child. In those studies that also reported before and after psychosocial measures in a population with successful treatment, data were analysed separately for treatment failures and treatment successes. Study populations needed to have undertaken at least one unsuccessful IVF or ICSI treatment cycle, including those with a treatment success.

Types of studies included were cohort studies and single-arm studies that employed pre- and post-treatment design methods. In the event of identifying multiple reports with the same patient population, the publication with the most inclusive and complete data was included, with reference made to all of the publications.

#### Exclusion

Studies were excluded if they did not contain pre and post psychological treatment scores or if there were insufficient data regarding these outcomes. They were also excluded if only published in conference proceedings, abstracts, or case reports. Studies for inclusion past the initial search were limited to those published in English. Refer to Prisma Checklist for further details (S1 Appendix).

#### Data extraction

A standardised form was used to extract data by one reviewer and checked by a second for accuracy and completeness (S2 Appendix). Uncertainty about inclusion of studies was resolved by discussion and consensus and data were only reported if they could be accurately extrapolated from the article. Reviewers were not blinded to authors, institutions, and journal of publication.

### Data synthesis and analysis

#### Meta-analysis

Studies that reported anxiety, depression or depressive symptoms pre and post ART treatment were included in the meta-analysis. The analysis investigated the effects of treatment on anxiety or depression in those with treatment failure, and—if data were available—also separately for those with treatment success. For each included study, the treatment effect was the difference between the pre and post therapy scores in either anxiety or depression (or depressive symptoms). Only complete pre and post data were included in the analysis.

To account for the differences between studies in the scales used to measure depression/depressive symptoms or anxiety, the standardised mean difference (SMD) between pre and post treatment groups was derived for each study and used to derive the subtotal and total estimates of the treatment effect [31].

The SMD was calculated as the mean difference between pre and post groups divided by the within-groups standard deviation of the assessment of anxiety or depression pooled across studies. A random-effects model using DerSimonian and Laird method [32] was employed to incorporate an estimate of the between-study variation into both the study weights and the standard error of the estimate of the common effect [31]. The precision of an estimate from each included study is represented by the inverse of the variance of the outcome pooled across all participants. Less precise estimates have larger variances, so the inverse of variance is smaller for studies with less precise estimates.

We introduced Hedge’s correction factor in order not to overestimate the value of the SMD calculated using the following formula:

$$J\left(df\right)=1-\frac{3}{4\text{df}-1}$$

where, *df* stands for the degrees of freedom used to estimate the within-groups pooled standard deviation [33].

The between-study heterogeneity was investigated using a random-effects meta-analysis regression which investigated the extent to which statistical heterogeneity between studies could be related to one or more characteristics of the studies [34]. Publication bias was evaluated using a funnel plot [35]. Some studies did not report a standard deviation (SD) for their study outcomes. For these we estimated a SD using the standard error, sample size and reported mean [31].

The meta-analyses were conducted by gender for depression or anxiety. Similarly, we conducted separate analyses by time period from the procedure. We defined ‘early period’ as up to five months from procedure, while ‘late period’ was defined as six months or more from procedure similar to the definitions of Freeman et al study [36].

#### Narrative analysis

The narrative synthesis reported on psychological outcomes other than depression or anxiety. The narrative synthesis was used to identify emerging patterns and to explore relationships within and between the studies [37]. Data were grouped according to psychological outcome, against treatment failure and treatment success if reported, and by gender. Results from sub analyses conducted by the included studies were reported if there were adequate data on outcomes associated with IVF or ICSI treatment and outcomes associated with women’s age.

#### Methodological quality of included studies

The quality of the studies included in both the meta-analysis and the narrative synthesis was assessed while addressing five possible sources of bias that related to: study participation; study attrition; measurement of prognostic factors; measurement of outcomes; and analysis approaches [38]. The scoring was based on and guided by Hayden et al [38] tool that evaluated the quality of prognostic studies in systematic reviews. The quality of these studies was independently checked by three researchers (co-authors AM, GM and an external assessor TDV) (S1 Table). Percent agreement was calculated together with Cohen’s Kappa coefficient that measured the inter-rater agreement.

## Results

### Included studies

**Fig 1:**
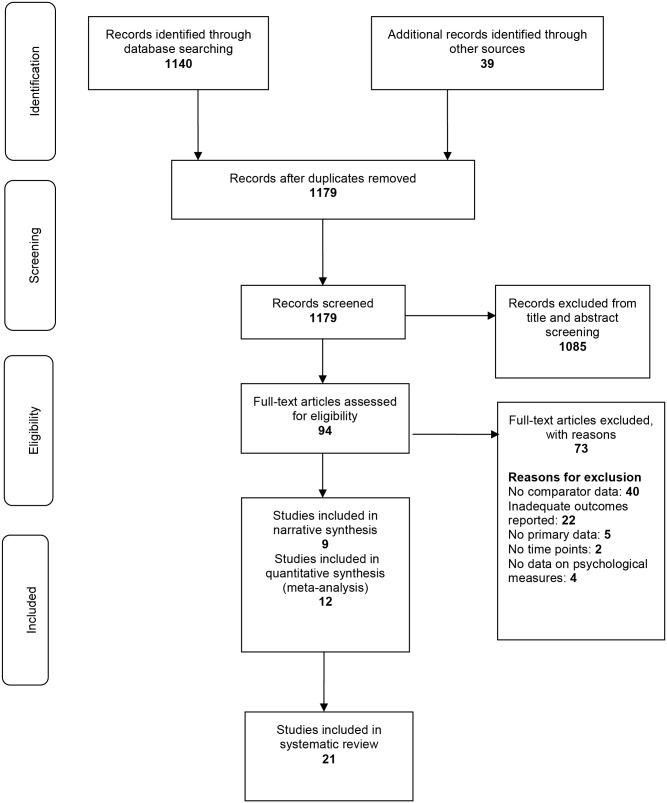
Study selection flow chart.

A total of 21 studies were included in the review from an initial search of 1140 records with an additional 39 identified from reference lists and other sources. A total of 94 papers were reviewed for full text. Following full text review, 73 studies were excluded (Fig 1).

The 21 studies in the systematic review focused on women, or couples who had experienced ART treatment failure or success within the same population. One study reported on men only. All included studies followed a pre and post treatment design with first date of publication 1992. Studies were conducted in nine different countries with The Netherlands contributing the largest number of papers from a single country. Study participants were recruited from hospitals and fertility clinics with a total population size of 7,258 women and 5,653 men. Of the studies that compared outcomes among groups by treatment success, 573 women had successful treatment while failed treatment was reported in 1,751 couples. The mean age for women ranged from 32 to 44 years and men 33 to 38 years. Infertility duration per study ranged from 2 to 8 years (Table 2).

**Table 2:** Study characteristics.

Reference country	Population (n =)	Age		Infertility duration (Mean, SD, Years)	Treatment		Psychological outcomes	Measure scales	Measurement at time points		
		Female (Mean, SD, Years)	Male (Mean, SD, Years)		1^st^ ART treatment	IVF or ICSI			Short (2 weeks to 5 months)	Mid (6 to 11 months)	Long (12 months or greater)
An et al; 2013 China^b^	Women (264)	Pregnant 33.1 (4.1)	NR	Pregnant 6.8 (3.3)	Y	IVF or ICSI	Anxiety	STAI	Y	Y	N
		Non-pregnant 33.4 (3.9)	NR	Non-pregnant 33.4 (3.9)			Depression	BDI	Y	Y	Y
Black et al., 1992;USA^a^	Women (88)	35.0	38.0	NR	NR	IVF	Distress	IFQ	Y	Y	N
							Stress	Likert scale			
Borneskog et al, 2014, Sweden^a^	Women (63) Men (63)	NR	NR	NR	NR	IVF	Relationship satisfaction	ENRICH	Y	N	Y
Holter et al., 2006; Sweden^b^	Women (117) Men (117)	32.2 (3.7)	33.9 (5.5)	4.4 (2.2)	Y	IVF or ICSI	Psychological wellbeing (anxiety, depression, anger, happiness etc)	PGWB	Y	N	N
Hynes et al.,1992;Aust^b^	Women (100)	32.0	NR	NR	NR	IVF	Depression	RDCD	Y	N	N
							Self esteem				
Khademi et al.,2005;Iran^b^	Women (251)	28.9 (5.5)	NR	6. 9 (4.5)	N	NR	Depression	BDI	Y	N	N
Leiblum et al., 1987; USA^b^	Women (59) Men (59)	33.0	34.0	4.5	N	IVF	Marital satisfaction	MAT	NR	NR	NR
							Mood (anger, depression, anxiety, vigor, fatigue)	POMS			
Lok et al., 2002; Hong Kong, China^b^	Women (372)	34.0 (3.4)	NR	4.5 (2.4)	N	IVF	Depression	BDI	Y	N	N
							Psychological wellbeing	GHQ			
Matthiesen et al., 2012; Denmark^b^	Women (45) Men (37)	32.2 (3.9)	NR	2.0 (1.2)	Y	IVF or ICSI	Mood (depression, anxiety)	POMS	Y	N	N
							Stress	COMPI Stress			
Newton et al., 1990; Canada^b^	Women (947) Men (899)	31.4 (3.8)	33.5 (4.9)	NR	Y	IVF	Depression	BDI	Y	N	N
							Anxiety	STAI			
							Life appraisal	LAI			
Pasch et al., 2012; USA^b^	Women (202)	35.5 (4.4)	NR	>2 years among 47.5%	Y	IVF	Depression	CES-D	N	Y	N
							Anxiety	STAI			
Peronace et al., 2007; Denmark^a^	Men (818)	NR	34.0 (5.0)	4.3 (2.4)	N	IVF or ICSI	Stress	SPQ	N	N	Y
							Mental health	SF-36			
							Coping effort	WOC			
Peterson et al., 2009; Denmark^a^	Women (1406) Men (1406)	32.0	34.4	4.0	Y	IVF or ICSI	Personal distress	COMPI	N	N	Y
							Marital distress				
							Social stress				
Peterson et al., 2011; Denmark^a^	Women (1406) Men (1406)	32.7 (3.5)	35.1 (5.0)	4.2 (2.3)	NR	NR	Marital benefit	COMPI	N	N	Y
							Coping				
Slade et al., 1997, United Kingdom^b^	Women (144) Men (144)	32.2 (3.4)	34.7 (4.6)	8.3 (3.0)	Y	IVF	Depression	BDI	Y	Y	N
							Mood (anxiety, confusion)	POMS			
Sydsjo et al., 2005; Sweden^a^	Women (320) Men (320)	32.0 (2.5)	33.6 (2.6)	4.0	Y	IVF	Marital dynamics	ENRICH & PCA	N	Y	Y
Verhaak et al., 2001; Netherlands^a^	Women (250) Men (250)	33.4 (3.7)	NR	3.7 (2.0)	N	IVF or ICSI	Anxiety	STAI	Y	N	N
							Depression	BDI			
							Mood	POMS			
							Marital satisfaction	MMQ			
Verhaak et al., 2005a; Netherlands^b^	Women (148) Men (71)	34.1	36.3	>5yrs 20%	Y	IVF or ICSI	State anxiety	STAI	Y	Y	N
							Depression	BDI			
Verhaak et al., 2005b The Netherlands^a^	Women (386)	34.3	NR	3.3	Y	IVF or ICSI	Anxiety	STAI	Y	N	N
							Depression	BDI			
Verhaak et al., 2007b; The Netherlands^a^	Women (450)	33.4 (4.1)	NR	NR	Y	IVF or ICSI	Anxiety	STAI	Y	Y	Y
							Depression	BDI			
Visser et al., 1994; The Netherlands^b^	Women (126)	44.1 (11.7)	NR	NR	Y	IVF	Anxiety	STAI	Y	N	N
							Psychological wellbeing	HSC			

All studies were pre experimental with a pre and post treatment design;

^a^ = Narrative synthesis;

^b^ = Meta-analysis

NR = not reported; N = no; Y = yes; F = female; M = male

Abbreviations: IFQ (Infertility Questionnaire); PGWB (Psychological General Well-Being Index); RDCD (Research Diagnostic Criteria for Depression); BDI (Beck Depression Inventory); MAT (Marital Adjustment Test); POMS (Profile of Mood States); GHQ (General Health Questionnaire); COMPI (Copenhagen Multicentre Psychosocial Infertility Fertility Problem); STAI (State Trait Anxiety Inventory); LAI (Life Appraisal Inventory); CES-D (Centre for Epidemiologic Studies Depression Scale); SPQ (Stress Profile Questionnaire); SF-36 (Short-Form-36 Inventory); WOC (Ways of Coping); PCA (Positive Couples Agreement); MMQ (Maudsley Marital Questionnaire); HSC(HopkinsSymptom-Checklist); ENRICH (Evaluating and Nurturing Relationship Issues, Communication and Happiness Inventory).

### Meta-analyses

Overall 12 studies were included in the meta-analyses—all reporting on depression, depressive symptoms, or anxiety pre and post ART treatment failure. Seven of these 12 studies also reported outcomes on those with a successful treatment. Of the original samples of 2,775 women and 1,327 men, pre and post treatment full data were available for 1345 women and 279 men among those whose treatment failed, and 385 women and 135 men among those whose treatment succeeded.

**Fig 2:**
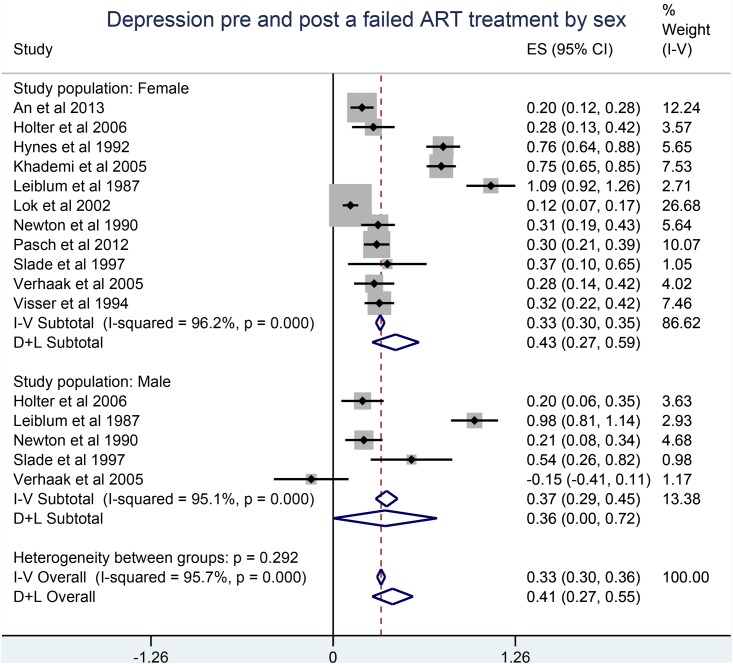
Depression pre and post a failed ART technology treatment by sex.

**Fig 3:**
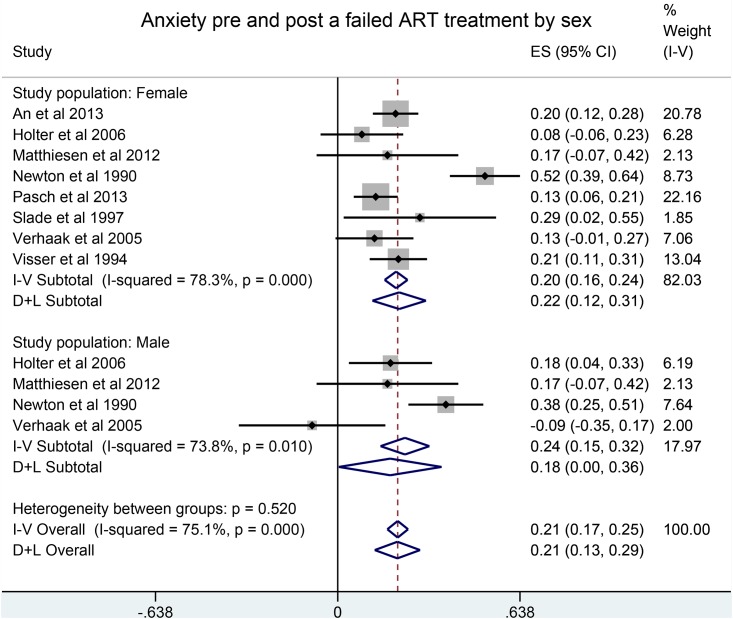
Anxiety pre and post a failed ART treatment by sex.

A failed ART treatment was positively associated with depression and anxiety in both males and females with an overall pooled SMD of 0.41 (95% CI, 0.27, 0.55) for depression (Fig 2) and 0.21 (95% CI: 0.13, 0.29) for anxiety (Fig 3).

**Fig 4:**
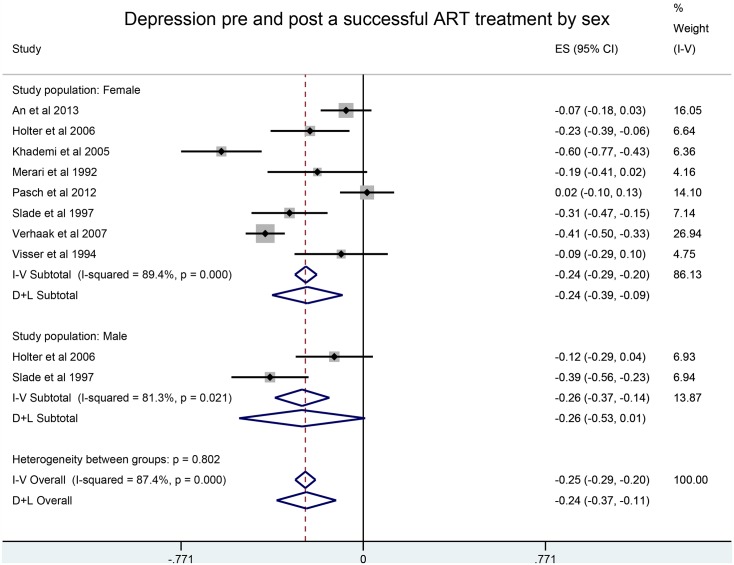
Depression pre and post a successful ART treatment by sex.

Results by gender were similar. In contrast, depression decreased after a successful treatment, SMD of -0.24 (95% CI: -0.37,-0.11) (Fig 4), whereas no statistically significant differences were observed between anxiety scores before and after a successful treatment.

Estimates and input parameters used to run the meta-analyses are shown in Table 3.

**Table 3:** Depression and anxiety pre and post a failed ART treatment in females: Standardised mean differences corrected to Hedges’ factor.

Study		Scale	Sample size	Mean (SD) before ART	Mean (SD) after ART	Standardised mean difference (variance of *d*)$d=\frac{\bar{Y1}-\bar{Y2}}{S\;within}$	*d* with Hedges’ correction (standard error)
**Depression**	An et al., 2013	BDI	172	1.6 (1.5)	1.9 (1.5)	0.200 (0.002)	0.199 (0.041)
	Holter et al., 2006	PGWB	50	1.7 (0.9)	2.0 (1.1)	0.282 (0.006)	0.278 (0.075)
	Hynes et al., 1992	SRMD	100	25.2 (4.3)	28.6 (4.5)	0.765 (0.004)	0.760 (0.060)
	Khademi et al., 2005	BDI	190	14.5 (9.8)	22.9 (11.8)	0.749 (0.003)	0.746 (0.052)
	Leiblum et al., 1987	POMS	59	6.4 (2.39)	9.39 (2.81)	1.103 (0.008)	1.089 (0.086)
	Lok et al., 2002	BDI	372	7.5 (6.8)	8.5 (8.6)	0.119 (0.001)	0.119 (0.028)
	Newton et al., 1990	BDI	151	4.6 (4.8)	6.4 (6.2)	0.311 (0.004)	0.309 (0.060)
	Pasch et al., 2012	CES-D	103	12.4 (10.2)	15.9 (11.7)	0.303 (0.002)	0.301 (0.045)
	Slade et al., 1997	BDI	14	8.3 (7.3)	11.1 (6.4)	0.398 (0.022)	0.374 (0.138)
	Verhaak et al., 2005	BDI	65	1.5 (2.3)	2.3 (2.9)	0.287 (0.005)	0.283 (0.071)
	Visser et al., 1994	HSCL	53	22.4 (9.2)	27.3 (13.1)	0.324 (0.003)	0.319 (0.052)
**Anxiety**	An et al., 2013	STAI	172	37.6 (10)	39.6 (7.6)	0.204 (0.002)	0.203 (0.041)
	Holter et al., 2006	PGWB	50	2.7 (1.1)	2.8 (1.2)	0.086 (0.006)	0.085 (0.074)
	Matthiesen et al., 2012	POMS-R	16	15 (9.1)	16.7 (9.4)	0.184 (0.018)	0.174 (0.127)
	Newton et al., 1990	STAI	149	32.9 (8.9)	39.1 (12.8)	0.518 (0.004)	0.516 (0.063)
	Pasch et al., 2012	STAI	103	41.4 (11.6)	43.3 (14.1)	0.134 (0.002)	0.133 (0.039)
	Slade et al., 1997	STAI	14	15.4 (7.4)	18.4 (2.8)	0.306 (0.021)	0.288 (0.136)
	Verhaak et al., 2005	STAI	65	37.3 (11.7)	39.0 (13.6)	0.130 (0.005)	0.129 (0.070)
	Visser et al., 1994	STAI	53	43.9 (11.4)	46.6 (12.7)	0.216 (0.003)	0.212 (0.051)

Abbreviations: ART (Assisted Reproductive Technologies); BDI (Beck Depression Inventory); BGWB (Psychological General Well-Being Index); CES-D (Centre for Epidemiologic Studies Depression Scale); C-STAI (Chinese State-Trait Anxiety Inventory); DACL (Lubin’s Depression Adjective Checklist); HSCL (Hopkins Symptom Checklist); POMS (Profile of Mood States); POMS-R (Profile of Mood States, Revised); SRMD (Self Report Measure of Depression); STAI (State Trait Anxiety Inventory).

Irrespective of ART outcome, both depression and anxiety became less prominent as time passed from procedure as shown in Table 4. Long term (i.e. six months or more from procedure) depression and anxiety were less than those measured shortly after the procedure (i.e. up to five months from procedure). Nonetheless, compared with baseline measures, those with a failed procedure continued to experience statistically significantly higher levels of depression and anxiety even after six months from the procedure.

**Table 4:** Early and late depression and anxiety by outcome of ART procedure.

Psychological measure	Time	Failed ART Effect size (95% CI)	Successful ART Effect size (95% CI)
Depression	Early	0.47 (0.29, 0.65)	-0.22 (-0.41, -0.03)
	Late	0.27 (0.11, 0.43)	-0.29 (-0.48, -0.10)
Anxiety	Early	0.25 (0.16, 0.34)	0.03 (-0.08, 0.14)
	Late	0.11 (0.02, 0.20)	-0.15 (-0.26, -0.04)

Early: Up to 5 months from procedure; Late: Six months and more from procedure.

The included studies were heterogeneous. The extent to which the study variables explained the heterogeneity in the treatment effects was evaluated using a random-effects meta-analysis regression showing that 54% of this heterogeneity was explained by year of study. An additional 20% of the heterogeneity was explained by the age of the woman. After adjusting for these two covariates, the remaining between-study variance was small, Tau-squared of 0.03. Baseline depression and gender did not contribute to the heterogeneity. Duration of infertility was not accounted for due to missing data. There was no evidence for a potential of publication bias as investigated by a funnel plot (results not shown).

### Narrative syntheses

The psychological outcomes in this synthesis were emotional adjustment and marital and relationship satisfaction for studies reporting both treatment failure and where evaluated, success within the same population. Full data for pre and post treatment scores were assessed.

### Emotional adjustment

#### Treatment failure

Emotional adjustment to treatment failure in couples varied with eight studies reporting negative outcomes. Adverse psychological outcomes ranged from more anger and less vigour experienced by couples, lower self-esteem in women, a decline in mental health status and an increase in psychiatric morbidity [39–42].

Overall women were found to have more negative emotional symptoms than men including higher emotional and distress levels, more frustration, powerlessness, and guilt, less contentment, less happiness, less confidence and lower satisfaction following failed treatment [43–45].

#### Treatment failure and success

Three studies reported psychological outcomes in groups experiencing different treatment outcomes. Women who were not successful felt more guilt, anger, frustration and isolation compared to those who succeeded [45]. Visser et al study [46] found that the psychological state of the women undergoing an IVF changed very little when pre and post treatment State-Trait Anxiety Index scores or Hopkins Symptoms Checklist scores were compared and observed in both those who had experienced a failed treatment, and those whose treatment had succeeded. Other outcomes reported more emotional distress experienced by unsuccessful couples six months after treatment cessation and negative mood increased in non-pregnant women compared to those with treatment success [46–48].

### Marital and relationship satisfaction

Marital benefit, marital adjustment, and sexual satisfaction were outcomes in eight studies with four of these reporting on treatment failure only.

#### Treatment failure

In the studies reporting on treatment failure, marital stress increased over time at 12 months and five years signifying the burden placed on their relationship [41, 44]. In contrast, IVF treatment *per se* positively impacted on marital relationships despite treatment failure. Women scored marital benefit positively compared to male partners, yet in another study women reported lower satisfaction in their relationship with their male partner [18, 49].

In a study by Sydsjo, scores on communication, relationships with family and friends and egalitarian roles were statistically significantly higher in couples after treatment failure than at baseline signifying a positive benefit [17]. However, gender differences showed that men had a poorer outlook on their relationship with statistically significantly lower results at each of three time points. In contrast, women found their sexual relationship and conception of life post treatment positively satisfying despite failure [17]. In comparison, one study reported that there was no difference in pre and post measurements for marital adjustment [39].

#### Treatment failure and success

Marital benefit, marital adjustment, and sexual satisfaction were outcomes in five studies. Couples who succeeded and those who did not, showed no differences in marital satisfaction [45]. Yet, relationship quality improved with successful treatment [46]. In contrast, unsuccessful couples adjusted poorly to marital and sexual relationships six months after treatment cessation [47]. Verhaak also found increased levels of sexual dissatisfaction in both groups despite the success in one group [48].

### Further analysis

#### Length of follow up and recovery

Most studies reported follow up of psychological outcomes within four weeks of unsuccessful treatment. In two studies, time predicted a statistically significant increase in marital distress and negative evaluation of their life situation [44, 50]. Other studies reported a decrease in adverse psychological symptoms illustrating some form of recovery post treatment failure. Over five years personal distress decreased and social distress did not change statistically significantly; after three years the relationship satisfaction decreased from measures taken prior to the start of treatment and at six month follow up, tension and fatigue reduced and vigour increased [44, 49, 47].

#### Number of treatment cycles

No difference in psychological outcomes was found after two or less cycles in depression or anxiety versus three or more cycles, while psychological difficulties increased between the second cycle for depression and dejection with vigour increasing. Results were similar in the third cycle but vigour decreased [51, 47].

#### Type of treatment

Only one study differentiated between IVF and ICSI and found no difference in the emotional reaction of women to treatment cycles by main effect of treatment type or interaction effect of treatment type with treatment outcome [48].

### Results of the quality assessment

Three raters assessed the quality of the studies included. AM assessed the quality of the studies included in the narrative synthesis; co-author GM assessed the quality of studies included in the meta-analysis; and the external assessor TDV assessed the quality of all studies.

Percent agreement between the assessors ranged from 75% to 79%. The combined Kappa coefficient for all three raters was 0.56 (95% CI 0.53–0.67) showing moderate agreement. These were similar when TDV ratings were compared with those with GM [Kappa 0.57 (95% CI 0.54–0.62)] or AM [Kappa 0.55 (95% CI 0.37–0.67)]. More detailed results of the quality check are shown in S2 Table.

## Discussion

The aim of this systematic review was to examine the evidence for associations between psychological outcomes and failed IVF/ICSI treatment. Most of the 21 studies in this review found psychosocial outcomes did negatively alter subsequent to failed ART treatment. A pooled estimate of the effect of failed treatment on psychological outcomes in twelve studies found higher depression and anxiety scores in women and higher scores for depression in men following failed treatment. Depression decreased after successful treatment.

It was difficult to ascertain if the study populations had one ART unsuccessful treatment cycle or a complete failure of a program of treatment; thus rendering an assessment of the potential association between prolonged failed treatment and increased adverse psychological outcomes unfeasible. Duration of infertility was not accounted for due to missing data. Similarly, available data did not permit us to run sub-analyses by different age groups. Finally, since the included studies followed a pre and post study design or a pre-experimental design with no controls, they had little power to establish causation.

Notwithstanding these limitations, results from meta-analysed studies and those included in the narrative syntheses are in accordance with studies that have described and reported results on the impact of treatment failure including depression, anxiety and infertility-specific distress [52–54].

Differences in gender with women experiencing more negative psychosocial outcomes have been reported elsewhere [55–56]. Similar to other studies, ours found marital dissatisfaction was associated with treatment failure and couples whose treatment succeeded faired psychologically better at post treatment [57–59]. Yet, in some of the included studies, failed treatment did not have a negative impact on marital relationship warranting further research in this area.

Our study revealed that although depression and anxiety become less pronounced as time passes from date of treatment (follow up post failed treatment was generally limited to short time points of less than five months), long-term depression and anxiety continued to be of concern. This is because compared with baseline measures, those with a failed procedure continued to experience higher levels of depression and anxiety even after six months from treatment. Other studies report varying effects suggesting either recovery post failed ART treatment or increased negative psychological adjustment deserving further research in this area [60–63].

The results from this study contribute evidence to a much broader debate around ART resource allocation and health policy decision making. In the absence of direct evidence of the impact of parental age on psychosocial outcomes, use may be made of indirect evidence examining psychosocial outcomes associated with failed or successful treatment. A linked evidence approach is used in health technology assessment to synthesise evidence systematically in order to contribute to the evidence-base for clinical decision making and clinical treatment effectiveness [64].

Previous work has revealed preconceptions among ART clinicians, consumers, and the broader community about the perceived positive psychosocial outcomes associated with having an opportunity to undergo ART [13, 65–66]. Hitherto, this consideration of the positive psychological effects of ART has overshadowed the evidence that negative psychological states are associated with treatment failure. Balancing the two is important for considerations around the indications of ART within a broader safety and effectiveness lens.

Linking ART success or failure and psychosocial outcomes may elucidate the experience of treatment subgroups, and how this might influence deliberations around recommendations for resource allocation and health policy. That negative psychological outcomes are associated with ART failure ought to feature more prominently in policy deliberations because, increasingly older couples are seeking ART treatment yet differential effectiveness leads to their markedly higher failure rates. ART failure is associated with increased depression and anxiety as highlighted in our study. Thus, ART treatment in older couples, compared with younger couples, is associated both with well documented reduced success in terms of live born infants and as demonstrated by indirect evidence, an increase in adverse psychological outcomes [5, 12].

## Conclusion

This is the first systematic review to conduct a meta-analysis with pre and post study designs in order to quantify differences in estimated effects between psychological scores before IVF/ICSI treatment and after treatment failure. Our study demonstrates the application of indirect evidence of psychological adverse outcomes within a more comprehensive decision-making framework for health policy around ART practice and resource allocation decision-making.

## Supporting Information

S1 Appendix PRISMA 2009 Checklist. (DOC)

S2 Appendix Data extraction form. (DOCX)

S1 Table Quality appraisal criteria. (DOCX)

S2 Table Accounting for potential bias as independently rated by three assessors. (DOCX)
