# Supplementary material for: Depression and Anxiety Outcomes Associated with Failed Assisted Reproductive Technologies: A Systematic Review and Meta-Analysis
Source: PLoS One. 2016 Nov 11;11(11):e0165805. doi: 10.1371/journal.pone.0165805 (PMC5106043; doi:10.1371/journal.pone.0165805)
Supplement: S2 Appendix — (DOCX) [file pone.0165805.s002.docx]

| **STUDY DETAILS**  Data extraction form | |
| --- | --- |
| **Reference:** | |
| **Location:** | |
| **Affiliation and source of funding:** | |
| **Study design:** | |
| **Number of Females:** | **Number of Males:** |
| **Female sample characteristics:**  Age: Mean, Median, SD:  Duration of infertility in years:  Infertility aetiology:  Duration of treatment:  Number of previous children: | **Male sample characteristics:**  Age: Mean, Median, SD:  Number of previous children: |
| **Selection criteria for females:**  Inclusion criteria:  Exclusion criteria: | **Selection criteria for males:**  Inclusion criteria:  Exclusion criteria: |
| **Treatment specifications:**  IVF or ICSI:  Donor or autologous oocytes:  Donor or autologous sperm:  Surrogate / gestational carrier: | **-** |
| **Number of treatment cycles:** | |
| **Follow-up and losses / withdrawals / treatment cessation:**  Duration of follow-up:  Withdrawals:  Lost to follow-up: [number, with reasons]:  Reasons for treatment cessation: | |
| **Outcome measures:**  Depression / depressive symptoms  Anxiety  Anger / Vigour  Self-esteem / confidence / communication problems  Mental health status / emotional distress / negative mood  Frustration / powerlessness / guilt  Contentment / happiness / general satisfaction  Problems relationships with family and friends  Marital / relationship satisfaction  Marital benefit / marital adjustment / sexual satisfaction | |
| **Data collection points in time pre and post treatment:** | |
| **Treatment outcome:**  Success  Fail | |
| **Type of failure:**  Total treatment failure:  Failure of a single cycle: | |
| **Treatment factors considered in study:** | |
| **Definitions and scales used:** | |
| **Statistical analysis conducted:** | |
| **Results for females:**  Pre-treatment depression measure  Post-treatment depression measure  Pre-treatment anxiety  Post-treatment anxiety  *Other reported outcomes:*  Marital/relationship satisfaction  Emotional adjustment to ART failure  Quality of life/health-related quality of life  Emotional wellbeing | **Results for males:**  Pre-treatment depression measure  Post-treatment depression measure  Pre-treatment anxiety  Post-treatment anxiety  *Other reported outcomes:*  Marital/relationship satisfaction  Emotional adjustment to ART failure  Quality of life/health-related quality of life  Emotional wellbeing |
| **Internal validity of study:** | |
| **External validity of study:** | |
| **Reviewer comments** | |
| **Extracted by:**  **Date:** | **Checked by:**  **Date:** |
