# Supplementary material for: Depression and Anxiety Outcomes Associated with Failed Assisted Reproductive Technologies: A Systematic Review and Meta-Analysis
Source: PLoS One. 2016 Nov 11;11(11):e0165805. doi: 10.1371/journal.pone.0165805 (PMC5106043; doi:10.1371/journal.pone.0165805)
Supplement: S1 Table — (DOCX) [file pone.0165805.s003.docx]

**Quality assessment of included studies**

Three assessors independently rated the quality of the studies included in both meta-analysis and narrative synthesis based on Hayden JA, et al 2006. The quality appraisal criteria are based on items listed in S1 Table. Confounding, which is also part of Hayden’s appraisal tool, was not assessed in our pre and post study designs because each participant was his/her control.

| S1 Table. Quality appraisal criteria. |
| --- |
| **Study population** |
| Source population clearly defined |
| Study population described |
| Study population represents source population or population of interest |
| **Study attrition** |
| Completeness of follow-up described |
| Completeness of follow-up adequate |
| **Prognostic factor measurement** |
| Prognostic factors defined |
| Prognostic factors measured appropriately |
| **Outcome measurement** |
| Outcome defined |
| Outcome measured appropriately |
| **Analysis** |
| Analysis described |
| Analysis appropriate |
| Analysis provided sufficient presentation of data |

Source: Hayden JA, Cote P, Bombardier C. Evaluation of the quality of prognostic studies in systematic reviews. Ann Intern Med 2006; 144:427-437.

**Results**

Percent agreement was calculated together with Cohen’s Kappa coefficient that measured the inter-rater agreement. The raters were asked to decide whether potential bias in each of the listed items in S2 Appendix, S2 Table was accounted for, partially accounted for, or not accounted for. Results are shown in S1 Appendix, S2 Table.

| S2 Table Accounting for potential bias as independently rated by three assessors | | | |
| --- | --- | --- | --- |
|  | **GM** | **AM** | **TDV** |
| Potential bias was accounted for in studies, % | 67.8 | 66.3 | 62.3 |
| Potential bias was partially accounted for in studies, % | 26.2 | 19.3 | 26.6 |
| Potential bias was not accounted for in studies / missing information, % | 6.0 | 14.4 | 11.1 |
| GM: Assessed the quality of studies included in meta-analysis  AM: Assessed the quality of studies included in narrative synthesis  TDV: Assessed the quality of all studies | | | |

The combined Kappa coefficient for all three raters was 0.56 (95% CI 0.53-0.67) showing moderate agreement. These were similar when TDV ratings were compared with those with GM [Kappa 0.57 (95% CI 0.54-0.62)] or AM [Kappa 0.55 (95% CI 0.37-0.67)]
