# Supplementary material for: Depression and Anxiety Outcomes Associated with Failed Assisted Reproductive Technologies: A Systematic Review and Meta-Analysis
Source: PLoS One. 2016 Nov 11;11(11):e0165805. doi: 10.1371/journal.pone.0165805 (PMC5106043; doi:10.1371/journal.pone.0165805)
Supplement: S2 Table — (DOCX) [file pone.0165805.s004.docx]

**Quality assessment of included studies**

**Results**

Percent agreement was calculated together with Cohen’s Kappa coefficient that measured the inter-rater agreement. The raters were asked to decide whether potential bias in each of the listed items in S2 Table was accounted for, partially accounted for, or not accounted for. Results are shown in S2 Table.

| S2 Table. Accounting for potential bias as independently rated by three assessors. | | | |
| --- | --- | --- | --- |
| **Potential bias** | **GM** | **AM** | **TDV** |
| Potential bias was accounted for in studies, % | 67.8 | 66.3 | 62.3 |
| Potential bias was partially accounted for in studies, % | 26.2 | 19.3 | 26.6 |
| Potential bias was not accounted for in studies / missing information, % | 6.0 | 14.4 | 11.1 |
| GM: Assessed the quality of studies included in meta-analysis  AM: Assessed the quality of studies included in narrative synthesis  TDV: Assessed the quality of all studies | | | |

The combined Kappa coefficient for all three raters was 0.56 (95% CI 0.53-0.67) showing moderate agreement. These were similar when TDV ratings were compared with those with GM [Kappa 0.57 (95% CI 0.54-0.62)] or AM [Kappa 0.55 (95% CI 0.37-0.67)].
